# Comparison of therapeutic effects of N-Acetylcysteine with pregabalin in improving the clinical symptoms of painful diabetic neuropathy: a randomized, double-blind clinical trial

**DOI:** 10.1186/s40842-024-00172-x

**Published:** 2024-04-19

**Authors:** Firozeh Sajedi, Arman Abdi, Maryam Mehrpooya, Vida Faramarzi, Younes Mohammadi, Fateme Sheida

**Affiliations:** 1 Department of Internal Medicine, School of Medicine, Hamadan University of Medical Sciences, Hamadan, Iran; 2 Department of Clinical Pharmacy, School of Pharmacy, Hamadan University of Medical Sciences, Hamadan, Iran; 3 Modeling of Noncommunicable Diseases Research Center, School of Public Health, Hamadan University of Medical Sciences, Hamadan, Iran; 4 Cancer Research Center, Hamadan University of Medical Sciences, Hamadan, Iran; 5 Gastrointestinal and Liver Diseases Research Center, Guilan University of Medical Sciences, Rasht, Iran

**Keywords:** Painful diabetic neuropathy, N-acetylcysteine, Pregabalin, Oxidative stress

## Abstract

**Objectives:**

Painful diabetic neuropathy (PDN) is highly prevalent and annoyingly in patients with diabetes. The aim of this study was to investigate the effects of oral N-acetylcysteine (NAC) compared to pregabalin in PDN.

**Methods:**

One hundred two eligible patients with type 2 diabetes and PDN were randomly recievied pregabalin (150 mg/day) or N-Acetylcysteine (NAC) (600 mg/ twice a day) for 8 weeks. Mean pain score, Sleep interference score (SIS), Patient Global Impression of Change (PGIC), Clinical Global Impression of Change (CGIC), and also, serum levels of total antioxidant capacity (TAC), total thiol groups (TTG), catalase activity (CAT), nitric oxide (NO), and malondialdehyde (MDA) were assessed at baseline and at the end of the study.

**Results:**

NAC was well tolerated in all patients. The decrease in mean pain scores and increase in SIS was similar between two groups. More improvement in PGIC and CGIC from the baseline was reported in NAC group. NAC, significantly, decreased serum levels of MDA, and NO, but increased TAC, TTG, and CAT. Pregabalin, significantly, decreased serum levels of MDA, and NO and increased TAC.

**Discussion:**

NAC is efficacious in alleviate symptoms of PDN which is probably related to its antioxidant effects.

**Trial registration:**

The research protocol received approval from the Ethics Committee of Hamadan University of Medical Sciences (IR.UMSHA.REC.1397.137). The trial registry URL and number in Iranian Registry of Clinical Trials (IRCT): https://www.irct.ir/trial/33313, IRCT20180814040795N2 (Registration date: 2019-01-21, Retrospectively registered)

## Introduction

Diabetes is one of the most common endocrine disorders, affecting approximately 6% of the world's population [1, 2]. Studies have shown that insulin resistance and relative insulin deficiency are involved in the pathogenesis of this disease, leading to hyperglycimia [3–5]. The direct and indirect effects of hyperglycemia on human arteries are a major cause of diabetes mortality. In general, complications of diabetes are divided into two categories: macrovascular complications (coronary artery disease, peripheral artery disease and stroke) and microvascular complications (nephropathy, neuropathy and diabetic retinopathy) [4, 6, 7].

One of the major complications of diabetes is diabetic neuropathy. Diabetic neuropathy is defined as the presence of signs and symptoms of peripheral nerve disorders in patients with DM after rejecting other possible causes [8, 9]. It is estimated that by 2030, approximately 472 million people worldwide will have diabetes, of which at least 236 million will have DPN [10]. In DPN, which is a sensory neuropathy, both small and large fibers are damaged and can cause negative symptoms such as loss of touch, vibration, pinprick, heat and cold and positive symptoms such as hyper sensitivity, paradoxical pain, in the form of gloves and socks. First the symptoms related to small sensory fibers, then large afferent sensory fibers and finally motor fibers are created and can cause muscle weakness [11].

Various treatments are used to relieve the symptoms of neuropathy. DPN treatment currently includes proper glucose control - lifestyle modification, the use of analgesics and neuroprotective drugs [12]. According to the European Federation of Neurological Societies guidelines (FENS), Duloxetine-Amytriptyline-Pregabaline-Gabapantin-Venlafaxine is the first-line treatment for DPN and opioid is the second-line treatment. Unfortunately the recommended pharmacological treatments are suboptimal [13].

Pregabalin is one of the most common drugs used to treat neuropathy, but high doses are not recommended due to dose-dependent side effects [14, 15]. Pregabalin has been used in many studies in the treatment of diabetic neuropathy and its effectiveness has been proven in comparison with placebo in the treatment of diabetic neuropathy [14, 16, 17]. The use of these drugs has limitations due to various side effects and drug interactions, especially in elderly patients with diabetes often take several other drugs for the underlying disease and most treatments are symptomatic.

Prolonged hyperglycemia in type 2 diabetes also increases oxidative stress, imbalance between oxygen and nitrogen free radicals, and this vicious cycle leads to the production of reactive oxygen species (ROS) [18]. Studies have shown that the most important source of ROS in hyperglycemic conditions is mitochondria and NADPH oxidase, leads to the production of predominantly superoxide anion during diabetes and the complications of diabetes [19]. Therefore, antioxidant are considered as one of the therapeutic compounds in the treatment of diabetic neuropathy [20]. N-acetylcysteine (NAC) has been proposed as a potential treatment for many diseases, like diabetic neuropathy, which oxidative stress is involved in their pathogenesis, both in animal studies and clinical trials [13, 20–23]. This is done by activating metabotropic glutamate receptor 2 (mGluR2). In a study using mice with a glucose above 250 mg / dlit induced by intraperitoneal injection of streptozocin, daily NAC injection for 7 days had analgesic effects [13].

In a clinical trial study conducted by Heidari et al. (2019), it was stated that N-acetylcysteine with antioxidant effect can reduce the pain of painful diabetic neuropathy [24]. In the Fishbane study, N-acetylcysteine was found to protect against oxidative tissue damage, and this protective property was directly related to the drug itself or to the secondary stimulation of glutathione production [25]. Horst et al. reported that daily intraperitoneal injection of 150 mg / kg of NAC for 3 to 10 days had analgesic effects by reducing the level of NO-metabolites in CCI-induced neuropathic rats [26]. Therfore, the antioxidant activity of NAC has been attributed to the rapid reaction with free radicals as well as the return of reduced glutathione.

There is no specific treatment for diabetic neuropathy, and many medications are used only to relieve symptoms and to prevent neuropathy from getting worse. According to existing literature based on the need for new treatment options, in this study, we investigated the effect of oral NAC (as monotherapy) on pain control and reduction of oxidative stress factors in patients with painful diabetic neuropathy. These effects were compared with the well-known pregabalin treatment.

## Patients and methods

### Participants

This study was performed as a randomized, double-blind clinical trial on patients with painful diabetic neuropathy referred to Imam Khomeini Clinic and Shahid Beheshti Hospital in Hamadan from August 2018 until June 2019. Patients were informed about the study aims and the confidential and anonymous data handling. The research was conducted with the permission of the ethics committee of Hamadan University of Medical Sciences under the numbers IR.UMSHA.REC.1397.137 and IR.UMSHA.REC.1397.138. Then it was registered in the Iranian Registry of Clinical Trials under the number IRCT20180814040795N2 (Registration date: 2019-01-21). The study population was patients with type 2 diabetes who complained of symptoms of diabetic neuropathy.

Participants, who signed the written informed consent, were randomly assigned to receive two different drugs. The randomization was provided by an independent statistician to ensure that groups were matched for age, sex, and body mass index (BMI) where possible. Both the study participants and the investigators were blinded to the study medication. To ensure the study's double-blindness, another individual, separate from the study's principal researcher, packaged the medications in white containers and labeled them with the numbers 1 and 2. All subjects were taken off any pain medication for two weeks before participating in the trial. In the pregabalin group, patients received 150 mg of pregabalin capsule twice daily for 8 weeks. In the NAC group, patients with diabetic neuropathy received 1,200 mg of NAC effervescent tablets twice daily for 8 weeks. After diagnosis by the relevant specialist and having the criteria for inclusion in the study and obtaining consent, the patients were randomly divided into 2 different groups of drugs. Objectives of the study, possible beneficial effects, possible side effects and how to use the drug were explained for patients participating in the study. A checklist containing demographic characteristics was recorded for all patients.

### Sample size and the inclusion and exclusion criteria

In this study, we employed a superiority design and utilized a two-tailed test with a type I error rate of 5% and a study power of 80%. To determine the required sample size, we performed a power analysis, assuming a 40% reduction in pain in the pregabalin group and a 70% reduction in pain in the N-acetylcysteine (NAC) group, as well as an attrition rate of 20%. Using this method, we calculated that a sample size of 51 participants per group would be sufficient to detect a statistically significant difference between the groups with a confidence level of 95%. Inclusion criteria were: diagnosed with type 2 DM for more than one year, age 30 to 70 years, hemoglobin A1C (HbA1C) less than 10%, stable antidiabetic treatment regimen more than 1 month and maintaining the same treatment regimen during the study, painful distal symmetrical and sensorimotor polyneuropathy attributable to DM more than 3 months, diabetic neuropathy with an NDS (Neuropathy Disability Score) greater than or equal to 6, a VAS score of at least 4 for pain and a NSS (Neuropathy Symptom Score) higher than or equivalent to 5, no type 1 diabetes and non-pregnancy and lactation. DPN was confirmed by NSS and NDS criteria [27]. The exclusion criteria were: neuropathy due to other reasons during the study, creatinine clearance (CLcr) less than 30 mL/min calculated according to the Cockcroft & Gault formula [28], taking other medications to relieve the symptoms of neuropathy, patients with acute and chronic inflammatory conditions, pregnancy or lactation or expecting to get pregnant during the study, medical, psychological, or pharmacological factors interfering with the collection or interpretation of study data, existence of leg ulcers, cerebrovascular disease and discopathy and various neuropathies, presence of any adverse effects resulting in patients’ intolerance or complications, the patient's unwillingness to continue the study, or people who have taken less than 80% of the study period.

### Data collection

The diagnosis of neuropathy in patients at the beginning of the study was based on Neuropathy symptom score (NSS) and Neuropathy disability score (NDS) criteria and the severity of neuropathy was determined based on VAS criteria. The VAS criterion is a horizontal ruler from a score of zero to ten, which a higher score indicating greater pain intensity. This pain intensity was re-evaluated at the beginning of the study, week 4 and end of week 8. Sleep interference scores were re-evaluated at the beginning of the study and 4 weeks and 8 weeks after treatment. The sleep score criterion was used to assess sleep intensity. Thus, the effect of neuropathic pain on sleep disorders is a 10-point scale, which is zero non-sleep disorders and the 10 most severe cases of sleep disorders.

Changes in the patient's symptoms were assessed by PGIC (Patient Global Impression of Change) [3] and CGIC [4] (Clinical Global Impression of Change) after the end of the study. These two represent the patient and physician evaluation of the change of symptoms after the end of the study, respectively, which number 1 shows the most improvement and number 7 shows the significant deterioration of symptoms compared to the time of onset the study. In the present study, it was evaluated 8 weeks after the end of the treatment. At the beginning of this study, and at the end of 2 months, 5 cc of blood samples were taken from the patients and examined for changes in the levels of oxidative stress factors. Samples were centrifuged for 10 min. Serum specimen was separated and stored at −70°C until completion of all the samples. All samples were assessed in duplicate. Catalase levels, thiol groups, lipid peroxidation, nitric oxide and total antioxidant capacity were measured by the kit of kiazist company in Germany at the beginning of the study and at the end of 8 weeks. Vital signs and examination and questioning regarding the adverse effects were performed at each of the 4-week follow-up visits. Compliance was assessed by direct questioning. The investigator was accessible by telephone to all patients throughout the study.

### Statistical analysis

The data was analyzed by statistician who was not aware of the intervention type assigned to each group, by using the SPSS version 16. Analysis of variance (ANCOVA), independent t-test and chi- square test were used to compare the mean data. Mean ± standard deviation (SD) was used to express continuous variables. Categorical variables were reported as percentages. Mean (±SD) of continuous variables was compared between two groups using independent t-test and the distribution of categorical variables between two groups was compared using Chi-squared or Fisher's exact test. To compare means of variables including mean pain score and mean sleep interference score between two groups over time, as dependent variables, and control of their baseline differences, General Linear Model (GLM) ANOVA repeated measure was used to analyze data. Because of the deviation from sphericity assumption, we used Greenhouse-Geisser correction to perform ANOVA results. Moreover, effect sizes were extracted from Partial Eta Square (PES). The significance level was considered to be less than 0.05.

## Results

In this study, which aimed to compare the effects of oral N-acetylcysteine and pregabalin on the blood levels of oxidative stress biomarkers and pain management in patients with diabetic painful neuropathy, 51 cases of painful neuropathy in type 2 diabetes under pregabalin treatment and 51 cases of painful neuropathy in type 2 diabetes treated with N-acetylcysteine were evaluated. In Table 1, the demographic and baseline variables were compared in the two pregabalin and N-acetylcysteine groups.

**Table 1:** Baseline and demographic variables of patients

**Variable**	**Pregabalin**	**N-acetylcysteine**	***P*-value**
Gender			
Male (%)	7 (13.7%)	9 (17.6%)	**0.59**
Female (%)	44 (86.3%)	42 (82.4%)	
Average age (year)	57 ± 9	55± 8	**0.22**
Average duration of diabetes (years)	7.75±5.5	4.8±8.25	**0.65**
Average duration of neuropathy (months)	19.9±19.8	20±13	**0.97**
Urea level (mg / dL)	22.0±11.3	22±7	**0.87**
Creatinine (mg / dL)	1.0±0.16	1.0±0.2	**0.36**
Hemoglobin A1C (%)	7.9±0.8	8±0.8	**0.54**
Triglyceride (mg / dL)	171.7±85.5	158.2±71.2	**0.39**
LDL (mg / dL)	100.8±27.2	87±22	**0.004**
HDL (mg / dL)	43±10	42.5±7.6	**0.81**

As shown in Table 1, there was no significant difference between the two groups with respect to age, sex, duration of neuropathy, urea level, Hemoglobin A1C, Triglyceride and HDL levels. Therefore, the two groups were similar in terms of these baseline and demographic variables. The only difference between the groups was observed at the LDL level, which was higher in the pregabalin group than in the N- acetylcysteine group (*P*= 0.004).

There was no significant difference between the two groups in terms of mean pain score and mean sleep score before and after intervention. As shown in Table 2, there was a statistically significant difference in the mean scores of pain and sleep before and after the intervention in both groups. Thus, N-acetylcysteine, like pregabalin, improved pain scores and sleep interference. According to this table, the frequency of patients who reported "very much improved" was slightly higher in the N-acetylcysteine group than in the pregabalin group, and no worsening of symptoms was reported in any of the groups. At the end of the study, the percentage of patients who had a very high improvement from the physician's point of view in the group receiving N-acetylcysteine and pregabalin was 43% and 41%, respectively, and no worsening of symptoms was reported in either group. The number of patients with complications in the pregabalin group was 23 cases (44.1%) and in the N-acetylcysteine group was 13 cases (25.5%). According to the Chi-Square test, there is a significant difference (*P* = 0.001) between the two groups, in terms of complications (Table 2). The most common complication in the pregabalin group was drowsiness and the most common one in the N-acetylcysteine group was nausea and vomiting.

**Table 2:** Comparison of clinical variables before and after the intervention in the studied groups

Group		Time to intervention	Pregabalin	N-acetylcysteine	*P*-value
Variable					
Mean pain score		Before	6.9±1.8	6.3±1.85	0.36
		fourth week	5.0±1.1	4.0±1.5	0.43
		Eighth week	2.3±1.4	1.5±1.5	
		*P*- value	<0.001	<0.001	
Mean sleep score		Before	4.82±2.2	6.11±2.2	0.43
		fourth week	6.9±1.9	8.0±1.2	0.95
		Eighth week	9.42±0.7	9.7±0.5	
		*P*- value	<0.001	<0.001	
PGIC (%)	1	-	40	42	-
	2	-	38	37	-
	3	-	20	20	-
	4	-	2	1	-
	5	-	0	0	-
CGIC (%)	1	-	41	43	-
	2	-	38	39	-
	3	-	19	17	-
	4	-	2	1	-
	5	-	0	0	-
Side effects (N (%))					
Drowsiness		After	11 (21.6)	0	
Nausea			3 (6)	8 (15.7)	0.001
Dyspepsia			1 (2)	5 (9.8)	
No side effects			28 (54.9)	38 (74.5)	

Each group was compared before and after the intervention in terms of paraclinical variables. In the first group, mean TAC (nmol/ ml), mean MDA (nmol/ ml) and mean NO (μM) were significantly different (*P* < 0.05). There was no significant difference (*P*> 0.05) between the two variables of mean TTG (mM) and mean CAT (munit/ ml). In the second group, all five variables had significant differences (Table 3).

**Table 3:** Comparison of paraclinical variables before and after the intervention in the studied groups

Group	Time to intervention	Pregabalin	N-acetylcysteine	*P*-value
Variable				
TAC (nmol/ ml)	Before	803.5±133.7	765±101.8	0.10
	After	818.9±95.7	855.2±102.1	0.15
	*P*- value	0.02	0.001	-
MDA (nmol/ ml)	Before	35.4±4.7	35.4±5.3	0.99
	After	32.85±4.5	30.21±6.5	0.07
	*P*- value	0.001	0.001	-
TTG (mM)	Before	343±39	346±50.2	0.72
	After	344.5±41.5	355.2±47.2	0.34
	*P*- value	0.25	0.04	-
NO (μM)	Before	66.8±3.62	66.8±3.7	0.97
	After	64.45±3.5	63.47±3.95	0.30
	*P*- value	0.001	0.001	-
CAT (munit/ ml)	Before	546.8±38.8	546.4±40.6	0.95
	After	541.2±41.6	556.6±42.7	0.15
	*P*- value	0.22	0.03	-

## Discussion

Considering the risk of developing diabetic neuropathy in patients with DM on one side and the role of oxidative stress in the pathogenesis of it on the other hand, in the present study we investigated the effects of oral NAC, as a drug antioxidant effects, in reducing the symptoms of painful diabetic neuropathy in patients with type 2 diabetes. Based on the results, the two treatments were equally effective in improving sleep and pain disorders due to painful neuropathy, but comparisons in each group before and after the intervention, showed significant improvement in sleep disorder and pain scores in both groups. The mean scores of PGIC and CGIC were almost similar in both groups. The NAC group showed a significant difference in all five studied oxidative stress variables before and after the intervention. Based on the above findings, it can be concluded that NAC with antioxidant mechanism can be used in the treatment of painful diabetic neuropathy. An interesting finding in our study was the antioxidant effect of pregabalin, which showed that perhaps one of the therapeutic mechanisms of pregabalin in the treatment of painful diabetic neuropathy is its antioxidant effect, which has already been shown in animal studies with increased catalase and glutathione peroxidase [29, 30], but no human study of this pregabalin mechanism had been performed.

It seems that target therapy with antioxidant compounds can be effective as one of the treatments of diabetic neuropathy. NAC, as an amino acid derivative of cysteine (cys) and due to its ability as a glutathione precursor, has antioxidant effects. Due to its cytoprotective role, NAC inhibits the cellular response in which ROS specifically plays a major role [31]. NAC-derived cysteine is desulfurized to generate hydrogen sulfide, which in turn is oxidized to sulfane sulphu species (SSS), predominantly within mitochondria. Sulfane sulfur species produced by 3-mercaptopyruvate sulphur transferase and sulfide:quinone oxidoreductase (SQOR), are the actual mediators of the immediate antioxidative and cytoprotective effects provided by NAC. Matrix metalloproteinase (MMP) -9 and MMP-2 play a key role in neuropathic pain due to their effects on maturation of inflammatory cytokines and stimulation of neural inflammation. Therefore, inhibition of MMP-2 may be suggested as a novel treatment for neuropathy. Because NAC is involved in inhibiting MMP, it has been suggested as a treatment [32].

Another antioxidant mechanism of NAC is due to the breakdown of thiolated proteins, and the release of free thiols produces better antioxidant activity than NAC [33]. In previous studies, the use of NAC as an adjutant with pregabalin in the treatment of PDN showed that mean pain scores and mean sleep interference score improved compared with pregabalin alone [24]. In this study, serum levels of biomarkers such as MDA decreased, but SOD, TAC, GPX and TTG increased. In the present study, NAC was studied alone in controlling patient's pain in order to reduce the number of drugs used by the patient and eliminate drug interactions and side effects of pregabalin as a limiting factor in the use of this treatment.

Although the findings are novel, there are several limitations that caution against making broad generalizations based on them. An initial limitation of this study is the limited sample size, mostly including female individuals. Further investigation with increased sample sizes and a more balanced gender representation is necessary. An additional limitation is the very brief duration of the intervention. Further study with extended follow-up periods is necessary to confirm our findings. PDN was diagnosed based on clinical assessment and nerve conduction studies were not performed, which could be considered in future studies. In addition, only two samples were collected from each patient at the initiation of treatment and after eight weeks, which limited an in-depth investigation and understanding of the biochemical alterations related to diabetic neuropathy and the assessment of therapy outcomes. At last, we employed a low dosage of NAC in our research and maintained a consistent dosage throughout the trial. It is possible that higher dosages of NAC, with gradual increases, could have potentially provided even more positive results.

## Conclusion

Due to the pathogenesis of diabetic neuropathy (inflammation and oxidative stress), anti-inflammatory and anti-oxidative agents such as N-acetylcysteine can reduce the pain of diabetic neuropathy by its antioxidant mechanism and could be one of the possible treatments for PDN, which needs further investigations. Also, one of the therapeutic mechanisms of pregabalin in the treatment of PDN can be with antioxidant mechanism.

## Data Availability

The data supporting the findings of this study are available from the corresponding author upon request (Email: firozehsajedi@gmail.com).
